# Reprogramming Microbial CO_2_-Metabolizing Chassis With CRISPR-Cas Systems

**DOI:** 10.3389/fbioe.2022.897204

**Published:** 2022-06-23

**Authors:** Hai-Yan Yu, Shu-Guang Wang, Peng-Fei Xia

**Affiliations:** ^1^ School of Environmental Science and Engineering, Shandong University, Qingdao, China; ^2^ State Key Laboratory of Microbial Technology, Shandong University, Qingdao, China; ^3^ Sino-French Research Institute for Ecology and Environment, Shandong University, Qingdao, China

**Keywords:** carbon dioxide, CRISPR, genome editing, cyanobacteria, acetogen, methanogen

## Abstract

Global warming is approaching an alarming level due to the anthropogenic emission of carbon dioxide (CO_2_). To overcome the challenge, the reliance on fossil fuels needs to be alleviated, and a significant amount of CO_2_ needs to be sequestrated from the atmosphere. In this endeavor, carbon-neutral and carbon-negative biotechnologies are promising ways. Especially, carbon-negative bioprocesses, based on the microbial CO_2_-metabolizing chassis, possess unique advantages in fixing CO_2_ directly for the production of fuels and value-added chemicals. In order to fully uncover the potential of CO_2_-metabolizing chassis, synthetic biology tools, such as CRISPR-Cas systems, have been developed and applied to engineer these microorganisms, revolutionizing carbon-negative biotechnology. Herein, we review the recent advances in the adaption of CRISPR-Cas systems, including CRISPR-Cas based genome editing and CRISPR interference/activation, in cyanobacteria, acetogens, and methanogens. We also envision future innovations *via* the implementation of rising CRISPR-Cas systems, such as base editing, prime editing, and transposon-mediated genome editing.

## Introduction

Anthropogenic emission of carbon dioxide (CO_2_) has driven an unprecedented high level of CO_2_ in the atmosphere, leading to an approximately 1.1°C increase in the average global temperature (Tollefson, 2021). This increase has reached an alarming level and has caused global and local climate issues. It also leaves a very small window to achieve the 1.5°C target settled in the Paris Agreement and reinforced in the UN Climate Conference in Glasgow (COP26). The atmospheric CO_2_ level must be lowered by a significant amount by controlling the emission of CO_2_ and sequestering CO_2_ from the atmosphere at the same time. In this endeavor, biotechnology provides promising routes. In one way, carbon-neutral biotechnology utilizes sustainable carbon sources (e.g., agriculture and forest wastes) to produce chemicals (e.g., ethanol, butanol, and 2,3-butanediol), alleviating the reliance on fossil fuels and reducing CO_2_ emissions (Liu et al., 2020). In another way, carbon-negative biotechnology directly consumes industrial or atmospheric CO_2_ for the bioproduction of fuels and value-added chemicals (Liu et al., 2020; Liew et al., 2022).

With metabolic engineering and synthetic biology, the inventory of products from biological routes has been greatly expanded, and the production and yield have been improved. For instance, the baker’s yeast *Saccharomyces cerevisiae* has been genetically engineered to convert lignocellulosic feedstock to bioethanol and chemicals, exhibiting the potential of carbon-neutral biotechnology (Wei et al., 2013; Sun et al., 2021). Recently, *S cerevisiae* has been engineered to utilize wasteful CO_2_ accumulated during lignocellulosic sugar fermentation by the installation of a CO_2_ fixation pathway, transforming the correlated biotechnology from a carbon-neutral process to a carbon-negative technology (Li et al., 2017; Xia et al., 2017). Inspiringly, Gassler et al. (2020) generated an engineered yeast *Pichia pastoris* capably of growing with CO_2_ and methanol, opening a new window for heterotrophic yeast to use one-carbon (C1) compounds as sole carbon sources. Similar enterprises have been made in *Escherichia coli*, and artificial autotrophic *E. coli* has been generated *via* the implementation of CO_2_ fixation pathways and adaptive laboratory evolution (Antonovsky et al., 2016; Gleizer et al., 2019; Flamholz et al., 2020).

Another biological path is to employ microorganisms that metabolize CO_2_ innately, such as photoautotrophic cyanobacteria and chemoautotrophs, including acetogens and methanogens. These organisms can use CO_2_ as a carbon source from either industrial waste gases or the atmosphere (Fackler et al., 2021). CO_2_-metabolizing microorganisms have shown great potential as microbial chassis, and industrial attempts have been made (Liu et al., 2020; Liew et al., 2022). Given the advances in synthetic biology, these microbes play more important roles on the path towards a sustainable future with enhanced CO_2_ utilization efficiency and an expanded spectrum of products. For instance, cyanobacterium *Synechocystis* sp. PCC 6803 has been modularly engineered to produce a high titer of 1-butanol, short/medium-chain carbohydrate, and lactate from CO_2_ (Liu X. et al., 2019; Shabestary et al., 2021; Yunus et al., 2022). Lately, a pioneer study conducted by LanzaTech, Inc. (Skokie, IL, United States) shows that *Clostridium autoethanogenum* can convert syngas (consisting of CO_2_, CO, and H_2_) to acetone and isopropanol, and a pilot-scale fermentation in a 125-L scalable reactor was demonstrated (Liew et al., 2022). These advances have validated the capability of CO_2_-metabolizing chassis in the fixation of CO_2_ and production of value-added chemicals, and these succuss illustrated the ever-increasing power of synthetic biology in biotechnology.

CRISPR-Cas systems, the bacterial and archaeal immune systems, have been repurposed as synthetic biology tools for gene editing and regulation (Knott and Doudna, 2018). They have been revolutionizing biotechnology in fundamental ways. Though still in its infant stage, multiple CRISPR-Cas-based synthetic biology tools have been developed for cyanobacteria, acetogens, and methanogens, driving the rising of novel biotechnologies based on CO_2_-metabolizing microbes. Herein, we summarize the current progress of CRISPR-Cas systems in genetically engineering microbial CO_2_-metabolizing chassis, especially cyanobacteria, acetogens, and methanogens, for the conversion of CO_2_ to biofuels and value-added products, and we discuss the challenges and future endeavors in developing more efficient synthetic biology tools.

## Microbial CO_2_ Metabolizing Organisms

Microbial CO_2_-metabolizing chassis, mainly autotrophic microorganisms, can use CO_2_ as a sole carbon source for catabolic and anabolic activities. Until now, six CO_2_ fixation pathways have been identified, among which the Calvin cycle and Wood-Ljungdahl Pathway are the most understood and applicable routes (Fuchs, 2011; Muller, 2019; Gleizer et al., 2020). Compared to the complementary physiochemical strategies, biological ways have advantages in forming carbon-carbon bonds with one-carbon (C1) building blocks using either solar energy or redox power from inorganic compounds, i.e., iron, sulfide, and ammonia, offering better opportunities for bioproduction by the microbes themselves or in combined biotic-abiotic processes (Li et al., 2012; Sakimoto et al., 2016; Jin et al., 2021).

Photoautotrophic organisms, such as microalgae and cyanobacteria, can fix CO_2_ with solar energy through the Calvin cycle and produce a large variety of organic compounds. Notably, efforts have been made to design and build engineered cyanobacteria for the production of biofuels (i.e., biodiesel, bioethanol, and isobutanol), value-added chemicals (Santos-Merino et al., 2019; Xia et al., 2019), and food-related products, such as starch (Luan et al., 2019). Besides the photoautotrophs, *Cupriavidus necator* (formerly *Ralstonia eutropha*), a facultative chemolithotroph, can grow on CO_2_ through the Calvin cycle as well with H_2_ or formate as the electron donor without light. *C. necator* has also been engineered as a novel chassis for bioproduction (Li et al., 2012; Panich et al., 2021). Chemoautotrophic organisms harboring the Wood-Ljungdahl Pathway can utilize CO_2_ and H_2_ anaerobically. As the key representatives, acetogens, especially strains from the class of Clostridia (e.g., *C. autoethanogenum*, *Clostridium ljungdahlii, Acetobacterium woodii*, and *Eubacterium limosum*), have been interrogated and engineered to utilize CO_2_ (with H_2_) or CO_2_-containing mixed gases (Muller, 2019; Fackler et al., 2021). Due to the requirement and capability of co-utilization of H_2_, acetogens can also be the bridge connecting bioproduction and “Power-to-Gas” technology, generating a novel nexus “Power-to-X” (Molitor et al., 2019; Mishra et al., 2020). Similarly, methanogens play important roles in different bioprocesses in various niches, such as in the gut, in soil, and in engineered systems (i.e., wastewater treatment facilities). They produce CH_4_ from CO_2_ and H_2_ or other one-carbon compounds, like methanol (Thauer et al., 2008; Zabranska and Pokorna, 2018). As methanogens are archaea, they typically possess unique industrial merits, including high tolerance to temperature and osmatic stress, making them advantageous CO_2_-metabolizing chassis.

## CRISPR-Cas Based Systems

CRISPR-Cas-based synthetic biology tools are repurposed from the bacterial and archaeal immune systems (Jiang and Doudna, 2017), and the innovations in CRISPR-Cas-based systems have been reshaping biotechnology in fundamental ways (Knott and Doudna, 2018; Pickar-Oliver and Gersbach, 2019). For instance, an artificial autotrophic *P. pastoris* was generated *via* the integration of six foreign genes and deletion of three innate genes with the CRISPR-Cas-based gene-editing tool (Gassler et al., 2020). Recently, CRISPR-Cas-based methods have also been deployed to upgrade carbon-negative bioprocess by manipulating CO_2_-metabolizing chassis. In this section, we focus on the adaption of different CRISPR-Cas systems for the perturbation of CO_2_-metabolizing microbes. We highlight achievements and challenges in cyanobacteria, acetogens, and methanogens.

## CRISPR-Cas-Based Genome Editing

**FIGURE 1:**
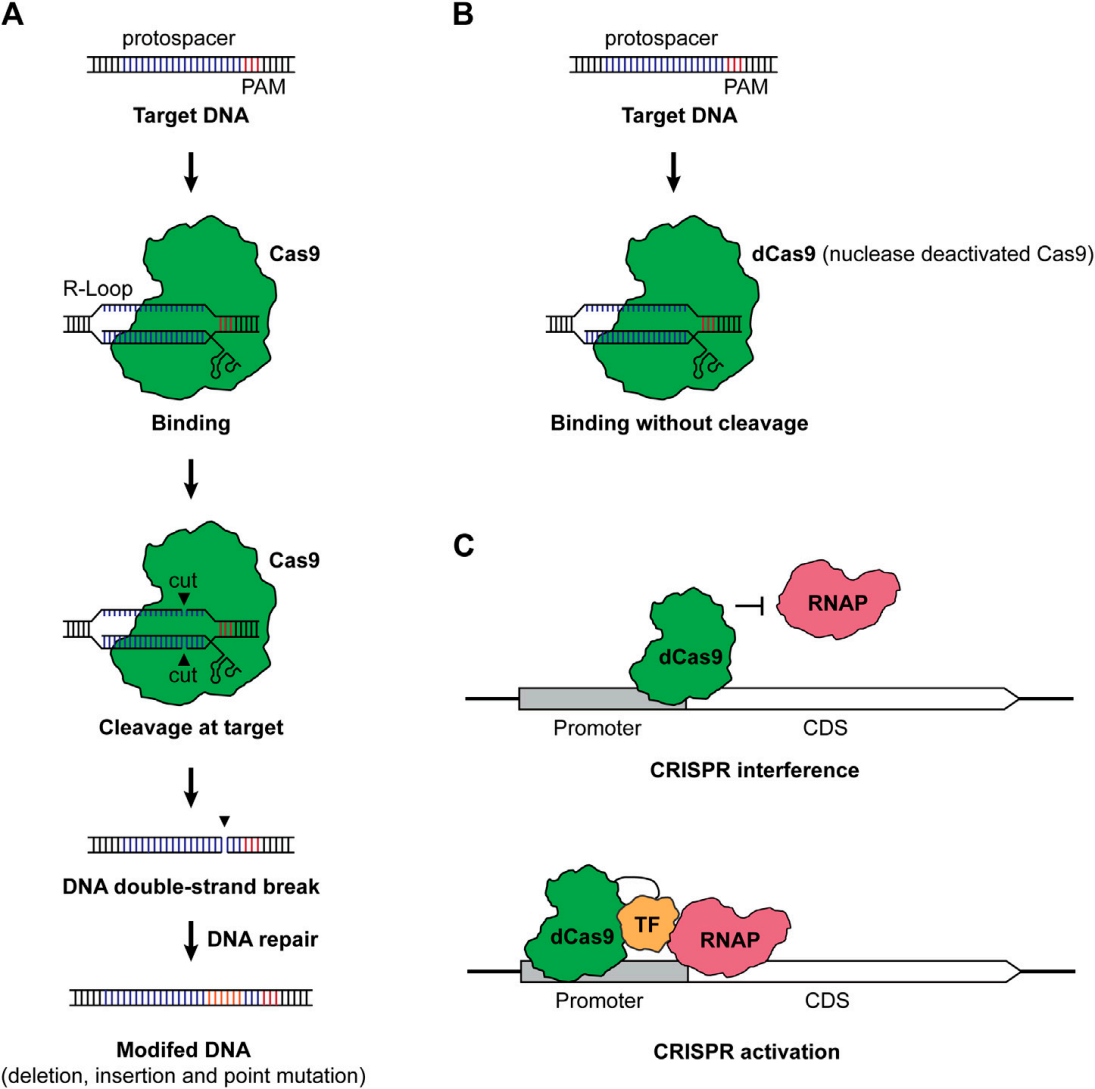
**(A)** CRISPR-Cas-based genome editing. Under the guidance of a gRNA containing a spacer complementary to the protospacer and a scaffold consisting of crRNA and tracer RNA, the Cas9 and gRNA complex finds and binds to the protospacer with a PAM, forming an R-loop. Then, Cas9 cuts the DNA in each strand, leaving a double-strand break (DSB) in the target. Finally, the target DNA will be modified with the repair of DSB. **(B)** Nuclease deactivated Cas9 protein (dCas9). When the nuclease activity of Cas protein was deactivated, the dCas9 still binds to the target sequence but it will not cleave the DNA anymore. **(C)** CRISPRi and CRISPRa. For CRISPRi, dCas9 binds to the promoter or coding region of a gene of interest and prevents the binding of RNAP, resulting in the repression of transcription. For CRISPRa, the dCas9-transcription factor (TF) fusion binds to the up region of the promoter. The TF helps recruit RNAP and allows the activation of transcription. We employed Cas9 from *S. pyogenes* as a representative in the figure, while a great variety of CRISPR systems can be used for genome editing, CRISPRi, and CRISPRa. TF stands for transcription factor, RNAP stands for RNA polymerase, CDS stands for coding sequence, and PAM stands for protospacer adjacent motif.

CRISPR-Cas-based genome editing in microbes typically has two steps: RNA-guided DNA cleavage and DNA repair of the double-strand break, the latter of which eventually resulted in the editing of a target gene (Selle and Barrangou, 2015; Knott and Doudna, 2018). Taking the Class II Type II CRISPR-Cas system from *Streptococcus pyogenes* as an example, the single CRISPR effector Cas9 is led by a guide RNA (gRNA) consisting of a targeting sequence (spacer), which is complementary to the target sequence (protospacer), a CRISPR RNA (crRNA) and trans-activating CRISPR RNA (tracer RNA) (Jiang and Doudna, 2017). When the complex of Cas9 and gRNA reached the target sequence, it recognizes the protospacer when a protospacer adjacent motif (PAM) presents. Then, the Cas9 nuclease cleaves the DNA and leaves a double-strand break, generating a “dead or alive” scenario for the microbe (Vento et al., 2019). With DNA repair mechanisms, the target gene will be edited to survive the deadly cleavage of Cas9 (Figure 1A). CRISPR-Cas-based genome editing has been prosperous due to the ease of use and the clean editing products without leaving a marker or a scar.

In cyanobacteria, CRISPR-Cas-based genome editing has been realized (Xia et al., 2019). Li et al. (2016) adapted a CRISPR-Cas genome editing tool for cyanobacterium *Synechococcus elongatus* PCC 7942 based on *S. pyogenes* Cas9 with a transient expression system. Later, a plasmid-based CRISPR-Cas system was developed for *S. elongatus* UTEX 2973, a fast-growing cyanobacterium showing great potential in sustainable bioproduction (Wendt et al., 2016). However, these countable successes implied the severe toxicity of Cas9 on cyanobacteria. Due to the toxicity of Cas9, conventional CRISPR-Cas-based genome editing tools with a “dead or alive” selection have not been thriving in cyanobacteria. To surmount this bottleneck, two strategies have been engaged. One is to use alternative Cas proteins. For instance, the Class II Type V CRISPR system with Cas12a as the effector showed lower toxicity than Cas9 to cyanobacteria. By using Cas12a, Ungerer and Pakrasi (2016) achieved CRISPR-Cas-based gene editing in *S. elongatus* UTEX 2973, *Synechocystis* sp. PCC 6803 and *Anabaena* sp. PCC 7102. Another way is to control the expression of the CRISPR-Cas system tightly. Hudson and colleagues hired a tightly regulated RNA device, the theophylline-responsive riboswitch, to maintain a low enough OFF-state expression of Cas9 to prevent its toxicity, and induce the genome-editing when required (Cengic et al., 2022). By applying this system, the reliable transformation of a replicable plasmid harboring CRISPR-Cas9 was obtained, leading to successful deletions and insertions of DNA fragments in the genome of *Synechocystis*. To our best knowledge, this study also reported multiplex genome editing in cyanobacteria for the first time regardless of methods. As multiple genes are typically involved in engineering a microbe for desired functionalities, multiplexing is of great importance in synthetic biology by saving considerable time and labor.

CRISPR-Cas-based genome editing tools have also been established in acetogens and methanogens. For acetogens, Cas9 and Cas12a based methods have been devised in *C. autoethanogenum* (Nagaraju et al., 2016)*, C. ljungdahlii* (Huang et al., 2016; Zhao et al., 2019), and *Eubacterium limosum* (Shin et al., 2019). To be noted, enhanced genome editing was achieved *via* a combination of CRISPR-Cas and serine recombinase (Huang et al., 2019). As reported, a phage serine recombinase was used for the integration of large DNA fragments while CRISPR-Cas inserts a small recognition motif of the recombinase. With this method, a butyric acid production pathway was successfully introduced to *C. ljungdahlii* for the production of butyric acid from syngas (Huang et al., 2019). Similar to the abovementioned method for cyanobacteria, a tightly regulated system controlled by a riboswitch, namely RiboCas, was designed to enable CRISPR-Cas-based genome editing in *Clostridium* strains, including *Clostridium pasteurianum*, *Clostridium difficile*, and *Clostridium sporogenes* (Canadas et al., 2019). Moreover, CRISPR-Cas-based deletion and integration were accomplished in methanogen *Methanosarcina acetivorans via* applying an inducible CRISPR-Cas9 system from *S. pyogenes* (Nayak and Metcalf, 2017). In the same study, the authors reported CRISPR-based deletion via the implementation of a foreign non-homologous end-joining (NHEJ) machinery from *Methanocella paludicola*, enabling the deletion of gene fragments (75–2.7 kb) without repairing DNAs. Recently, CRISPR-Cas-based genome editing was reported in methanogenic archaea *Methanococcus maripaludis* with the Cas12a system from *Lachnospiraceae bacterium* (Bao et al., 2022), further expanding the genome editing tools for methanogens.

## CRISPR Interference (CRISPRi) and CRISPR Activation (CRISPRA)

Actually, the toxicity of Cas proteins is not an exclusive issue in cyanobacteria, while most bacteria suffer from the toxic effects of CRISPR-Cas systems (Banno et al., 2018; Canadas et al., 2019; Vento et al., 2019). Despite screening alternative CRISPR-Cas systems and implementation of fine control modules, one alternative way is to employ the nuclease deactivated Cas protein (dCas) (Figure 1B). When the nuclease activity of Cas proteins is dead (dCas) or partially dead (nCas), dCas or nCas proteins are less toxic to bacteria compared to fully functional Cas effectors. By using dCas9, the resulting CRISPR-Cas system will no longer cleave the target DNA sequence but bind to the target. When dCas9 binds to the promoter or the coding sequence of a gene of interest, it will prevent the binding of RNA polymerase (RNAP), thus silencing the target gene at the transcription level and generating the method CRISPRi (Figure 1C) (Larson et al., 2013; Qi et al., 2013).

Due to the alleviated toxicity, CRISPRi obtained more popularity and has been developed for cyanobacteria. For instance, Yao et al. (2016) employed dCas9 to enable multiplex CRISPRi in *Synechocystis* sp. PCC 6803, Choi and Woo (2020) demonstrated CRISPRi with dCas12a in *S. elongatus* PCC 7942, and a dCas12a-based CRISPRi system was also established for *S. elongatus* UTEX 2973 (Knoot et al., 2020). In the former two reports, multiplex CRISPRi was achieved, and up to four genes were repressed at a single time, showing the huge potential of CRISPRi in engineering cyanobacteria for sustainable production (Yao et al., 2016; Choi and Woo, 2020). Notably, a CRISPRi system was devised for generating a gene repression library in *Synechocystis* sp. PCC 6803 in order to interrogate the genotype-phenotype interactions. By doing so, an industrially relevant strain with higher production of lactate, as a proof of principle, was engineered via CRISPRi-based repression of correlated essential genes (i.e., *gltA* and *pcnB*) related to lactate synthesis (Yao et al., 2020). More recently, CRISPRi was programmed as a genetic switch between cell growth and product synthesis. Shabestary et al. (2021) employed CRISPRi-based *gltA* regulation in a lactate-producing *Synechocystis* and achieve a high yield of lactate by decoupling cell growth and lactate production.

CRISPRi displays potential for acetogens and methanogens as well (Dhamad and Lessner, 2020; Fackler et al., 2021). Woolston et al. (2018) developed an inducible dCas9-based CRISPRi system for the repression of essential genes related to carbon metabolism in *Clostridium ljungdahlii*. Specifically, the *pta* gene encoding the phosphotransacetylase and the *aor2* gene encoding the aldehyde:ferredoxin oxidoreductase were repressed with CRISPRi individually and in a multiplex mode, redirecting carbon from acetate to the desired product 3-hydroxybutyrate with significantly increased titer and yield (Woolston et al., 2018). In a pioneer study, a CRISPRi system was developed for archaeal methanogen *M. acetivorans* by applying *S. pyogenes* dCas9, and the system was evaluated by interrogating the gene cluster related to nitrogen fixation (*nif* operon) and its regulator (*nrpR1*) (Dhamad and Lessner, 2020).

Besides gene repression, transcriptional activation can also be possible with CRISPR activation (CRISPRa) (Bikard et al., 2013; Liu Y. et al., 2019). CRISPRa deploys a combination of dCas protein and transcription factor, such as the ω subunit of the RNAP. When CRISPRa targets the upstream of a promoter, it will help bring RNAP and activate the transcription of the corresponding gene (Figure 1C). Bikard et al. (2013) first developed a CRISPRa system for bacteria and employed the dCas9-ω fusion to allow upregulation of *gfp* and *lacZ* expressions in *E. coli*. Recently, more advances in CRISPRa have been reported in bacteria (Liu Y. et al., 2019; Schilling et al., 2020; Kiattisewee et al., 2021; Villegas Kcam et al., 2021; Tickman et al., 2022). Given these advances, new transcriptional factors have been systematically screened, and the application has been expanded from *E. coli* to other bacteria, including *Paenibacillus polymyxa* (Schilling et al., 2020) and *Pseudomonas putida* (Kiattisewee et al., 2021). Moreover, a full range of gene regulation from repression to upregulation has been achieved by programmable CRISPRi/a circuits (Tickman et al., 2022) and by designing the targeting loci (Liu Y. et al., 2019). Though not been adapted yet, the application of CRISPRa and CRISPRi/a circuits will be a powerful tool for gene regulations in CO_2_-metabolizing microbes.

## Rising CRISPR-Cas Systems

### Base Editing

**FIGURE 2:**
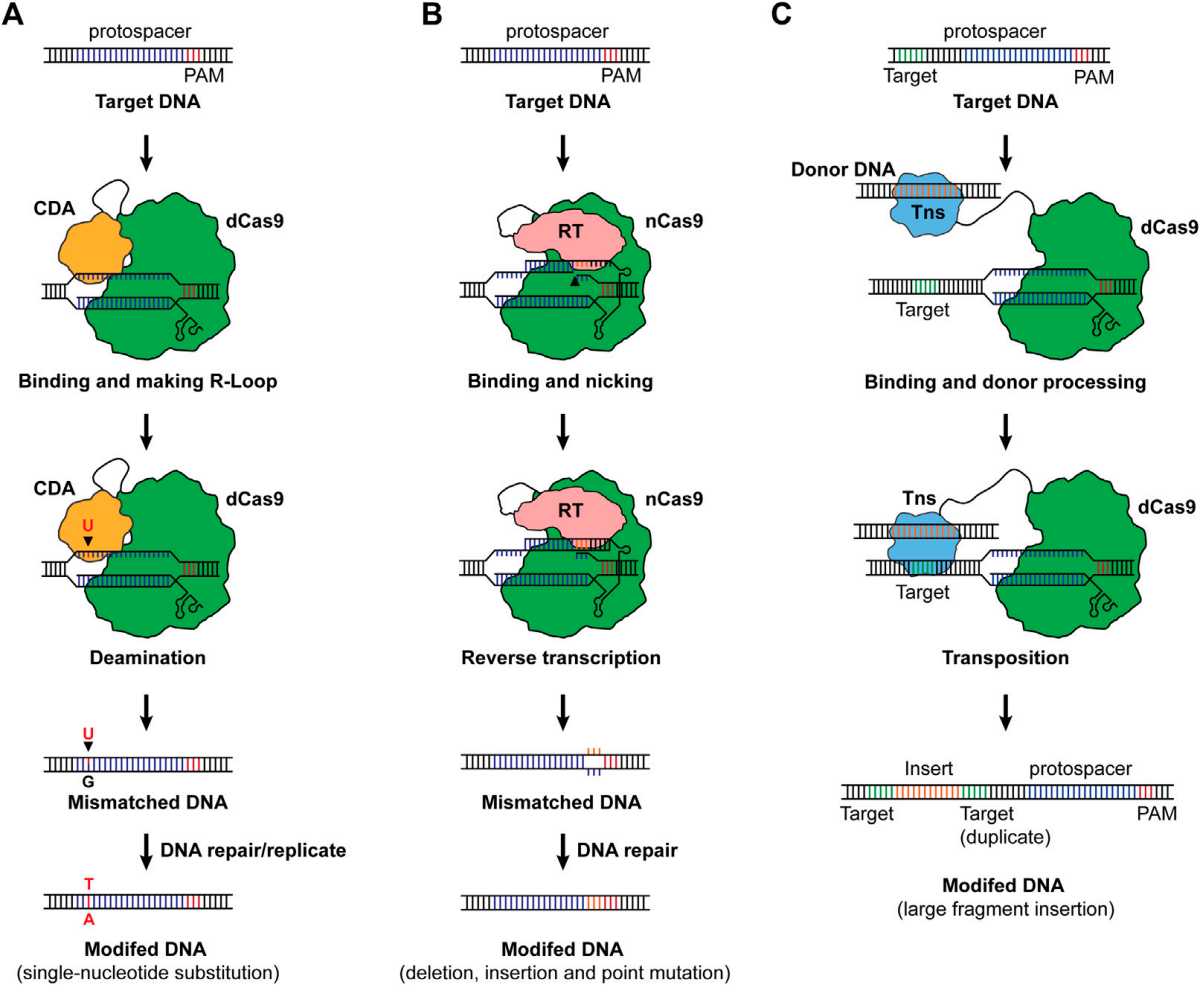
**(A)** Base editing. The cytosine base editor with a combination of dCas9 cytosine deaminase is shown as an example to illustrate the working mechanism. When dCas9 binds to its target sequence and an R-loop is formed, the cytosine deaminase (CDA) mutates a cytosine (C) to uracil (U), generating a uracil-guanine (U–G) mismatch. The C will then be replaced by thymine (T) along with the reparation of the mismatch upon DNA repair or replication, resulting in a C-to-T substitution at a one-nucleotide resolution. For adenine base editors, an A-to-G substitution will occur as a result of base editing. Notably, nCas9 can also be used as the effector for base editors. **(B)** Prime editing. The prime editing takes advantage of nCas9 and a fused reverse-transcription (RT). By designing a pegRNA consisting of the spacer for targeting, RT template with designed edits, and primer binding site (PBS), deletions, insertions and point mutations can be achieved. **(C)** Transposon-mediated CRISPR-Cas system for DNA integration. As a paradigm, dCas9 was fused to a transposase (Tns), and when the transposase was led to the target sequence, it recognizes the motif for transposition and integrates the donor DNA into the upstream of the protospacer, enabling large fragment insertions. Cas9 was used as the Cas effector for illustration of the basic working principles of base editing, prime editing and transposon-mediated editing.

A novel CRISPR-Cas-based genome editing was invented via combining nucleotide deamination, namely base editing (Figure 2A). For base editing, a dCas9 or nCas9 was fused with cytosine deaminase or adenine deaminase, and when binding to a target sequence, the deamination generates a mismatched base pair which will be repaired, resulting in C-to-T or A-to-G substitution (Komor et al., 2016; Nishida et al., 2016; Gaudelli et al., 2017). Along with emerging base editing methods, the single nucleotide substitution has been expanded to more combinations including C-to-G and C-to-A (Kurt et al., 2021; Zhao et al., 2021). Base editing was demonstrated in bacteria and exhibited prodigious capacities in engineering microbes for designed functionalities (Tong et al., 2019; Cheng et al., 2020; Rodrigues et al., 2021; Wu et al., 2021). Until now, one attempt reported the development and application of base editing in the chemoautotrophic acetogen. Xia et al. (2020) designed a C-to-T base-editing method for *C. ljungdahlii* using dCas9 and the activation-induced cytidine deaminase from the sea lamprey *Petromyzon marinus*, enabling precision genome editing at a one-nucleotide resolution. By applying this method, the carbon flux in *C. ljungdahlii* was redirected from ethanol to acetate, leading to increased production of acetate from CO_2_ and H_2_. Besides the common merits of CRISPR-Cas systems, base editing exhibits unique advantages in engineering CO_2_ metabolizing microbes in that: 1) the core module of base editing can be dCas or nCas which are less toxic to the host, 2) the system does not need repairing DNAs (donor DNA), and 3) it does not require high transformation efficiency to screen a survival cell from the direct “dead or alive” selection (Molla and Yang, 2019; Gu et al., 2021). These advantages make base editing a promising candidate for genome editing in CO_2_-metabolizing chassis.

### Prime Editing and Transposon-Mediated Integration

Despite the merits of base editing, the precision feature of base editing limits its capability in the deletion and insertion of DNA fragments. As a one-step forward, prime-editing was invented via a combination of nCas9 and a reverse-transcriptase, allowing the insertion of small DNA fragments without generating DNA double-strand breaks or requiring a donor DNA (Anzalone et al., 2019) (Figure 2B). Tong et al. (2021) further adapted this method for bacteria, making possible the introduction of deletions, insertions, and nucleotide substitutions with prime editing in *E. coli*. More specifically, an up to 97 bp of DNA fragment was deleted, and an up to 33 bp of DNA fragment was inserted into the genome of *E. coli* with high fidelity and efficiency (Tong et al., 2021). To enable large DNA insertion, a more recent study invented a twin-prime system with a prime editing system and serine recombinase. This system first inserts two motifs using the twin prime editing systems, and then the motifs will be recognized by the serine recombinase. Upon activation of the serine recombinase and the presence of a donor DNA, the DNA fragment can be integrated into the genome (over 5,000 bp of DNA fragment) or be inverted (up to 40 kb of DNA fragment) (Anzalone et al., 2021).

Another CRISPR-Cas-based tool for insertion of large-DNA-fragment is the transposon-mediated integration (Klompe et al., 2019; Strecker et al., 2019) (Figure 2C). Strecker et al. (2019) discovered a CRISPR-associated transposase from *Scytonema hofmanni* containing a Tn7-like transposase and a Type V-K Cas protein (Cas12k) and achieved insertion of 60–66 bp DNA fragment. Another work designed a system with dCas9 and Tn7-like transposon, enabling the integration of DNA fragments. The results show that the system is able to insert 1,000 bp of fragments with maximum efficiency, and efficient integration could also be achieved with a larger fragment (Klompe et al., 2019). Given these advances, prime editing and transposon-mediated integration, though not been realized yet, may offer powerful synthetic biology tools for genome editing in CO_2_-metabolizing microorganisms.

## Concluding Remarks

In this review, we summarized recent advances in developing and applying CRISPR-Cas systems for CO_2_-metabolizing chassis. CRISPR-Cas-based genome editing and CRISPRi, have been reported in these microbes, and the methods have been advancing biorefinery and bioproduction with CO_2_ as the carbon source, exhibiting great potential in alleviating CO_2_ emissions and in reducing atmospheric CO_2_ levels. However, more efforts are imperative to awake the full power of CRISPR-Cas systems in these CO_2_-metabolizing chassis. CRISPRa, base editing, prime editing, and transposon-mediated integration may offer encouraging future directions in developing novel CRISPR-Cas systems for CO_2_-metabolizing microorganisms. Moreover, discoveries of new CRISPR-Cas systems with special properties (e.g., a thermostable Cas9) are needed to engineer CO_2_-metabolizing microorganisms, such as thermophilic strains *Thermoanaerobacter kivui* and *Methanothermobacter thermautotrophicus* (Moon et al., 2019; Fink et al., 2021).

Besides CO_2_, CO, methane, methanol and formate are also important greenhouse gases and C1 compounds that can be obtained from waste gases and products or byproducts of clean energy industries. As such, natural and engineered C1-metabolizing microbes, including but not constrained in the autotrophs discussed here, will also be favorable microbial chassis for sustainable bioproduction. The development of novel synthetic biological tools, such as CRISPR-Cas systems, for C1 metabolizing organisms, will significantly foster innovations in carbon-negative biotechnologies.
